# Tissue Parathyroid Hormone Washout: A Reliable Method for the Localization and Exclusion of Parathyroid Adenomas

**DOI:** 10.7759/cureus.45814

**Published:** 2023-09-23

**Authors:** Elif Güneş, Nizameddin Koca, Fatih Hakan Tufanoğlu

**Affiliations:** 1 Department of Endocrinology, Metabolism and Diabetes, Health Sciences University, Bursa State Hospital, Bursa, TUR; 2 Department of Internal Medicine, Health Sciences University, Bursa State Hospital, Bursa, TUR; 3 Department of Radiology, Health Sciences University, Bursa State Hospital, Bursa, TUR

**Keywords:** ultrasound-guided tissue fine-needle aspiration with parathormone washout, parathyroid hormone washout, parathyroid hormone, specificity, sensitivity, parathyroid adenoma, parathyroid scintigraphy, primary hyperparathyroidism

## Abstract

Background and objective

Accurate identification of parathyroid adenoma (PA) is essential for minimally invasive treatment of primary hyperparathyroidism (PHPT). The aim of this study is to evaluate the results of parathyroid hormone (PTH) assay in aspirates of suspicious neck lesions and to clarify its reliability in determining whether the lesion is of parathyroid origin.

Methods

A total of 134 lesions (104 imaging-suspected PA and 30 concomitant thyroid nodules as a control group) of 101 patients were retrospectively analyzed. Patients with positive, negative, or never scintigraphy were included in our study. Ultrasound (US) was performed again and US-guided tissue fine needle aspiration with PTH washout (PTH-WO) was performed from suspicious lesions. A PTH-WO level higher than the patient's serum PTH levels is proposed for a positive test, and a PTH-WO level lower than the upper limit of the laboratory PTH level is proposed for a negative test. A definitive diagnosis was made postoperatively histopathologically.

Results

PTH-WO levels (median (IQR)) were significantly higher in the positive group (n = 93, 5000 (1600) ng/L) compared to the negative group (n = 11, 17 (13.1) ng/L) and thyroid nodule aspirate group (n = 30, 14 (4.3) ng/L) (p < 0.001). Among 93 PTH-WO-positive lesions, 42 lesions (45.1%) were not identified by parathyroid scintigraphy (PS), 20 (21.5%) lesions were suspiciously PS, and 33 lesions (35.4%) were negative or suspicious by first US findings. Of the 93 patients localized with PTH-WO, two were cystic PAs ablated by aspiration. The final pathology result of 91 operated patients proved PA. The sensitivity, specificity, negative predictive value, and positive predictive value of the PTH-WO were observed as 1.00, 0.82, 0.09, and 0.91, respectively.

Conclusion

Comprehensive repeated US and PTH-WO from the lesion increase the accurate localization of PAs. PTH-WO is a highly reliable method for differentiating parathyroid lesions from other cervical lesions.

## Introduction

Primary hyperparathyroidism (PHPT) is the third most common endocrine disorder [1], with an estimated prevalence of 176 cases in women and 77 cases in men per 100,000 people per year (1995-2010) [2].

Bilateral neck exploration (BNE) was the standard treatment for PHPT previously; however, this method requires the examination of all four parathyroid glands, even for a single parathyroid adenoma (PA) [3]. Focused parathyroidectomy (FP) is designed to limit surgical intervention to only the PA region. When the preoperative localization of PA is correctly performed, it has a high success rate like BNE, and FP reduces the operation time and complications, resulting in greater patient satisfaction and, from the perspective of the physician and healthcare team, less labor loss [4,5]. On the other hand, non-surgical treatment methods such as ultrasound (US)-guided radiofrequency ablation (RFA), a less invasive technique, have recently been used in PA treatment [6,7]. Its success rate is directly proportional to the accuracy of the adenoma localization.

Although parathyroid scintigraphy (PS) and US are the most frequently used imaging modalities for localizing PAs prior to surgery, they are insufficient for distinguishing parathyroid lesions from other lesions [8-10]. Their specificity and sensitivity are insufficient and vary across studies [11,12]. Inflammatory thyroiditis, thyroid lesions, lymph nodes, and malignancy of this region may cause false-positive results, whereas small PAs, multi-glandular disease (MGD), and cystic lesions may lead to false-negative results [13,14].

Even with four-dimensional computed tomography (4D-CT), it is only partially useful since it is not widely accessible [15]. Although PA is usually found in accessible areas, cytology alone is not helpful in the diagnosis of most cases and is therefore not used as a diagnostic test for PAs, unlike in thyroid lesions [16,17].

None of the imaging techniques can definitively direct the surgeon to FP; they can only guide the surgeon to the possible locations. Furthermore, when examining PA, 70% of patients with discordant or negative imaging findings still have uniglandular disease; thus, these cases should be handled carefully until the end, as the main aim when trying to localize PA is to prepare patients for a successful treatment process [18,19].

In patients with PHPT, US-guided fine needle aspiration (FNA) of the suspicious lesion and parathyroid hormone (PTH) washout (PTH-WO) was first used by Doppman and colleagues in 1983 [20]. PTH-WO, which has high sensitivity and specificity, is an accurate indicator of the parathyroid tissue and performs better than PS or US alone [21,22]. Thus, it can accurately guide the physician preoperatively and replace secondary procedures, such as intra-operative PTH (IO-PTH) monitoring [23]. The use of FP in combination with this confirmatory method in PA diagnosis is increasing [24,25].

Due to their distinct nature, these endocrine glands can be found in many locations in the neck, even in unexpectedly diverse regions. There is no imaging technique that definitively shows that the lesion originates from the parathyroid gland. This study aimed to assess the reliability of the US-guided PTH-WO method in PA localization, which is not routinely used for localizing parathyroid lesions.

## Materials and methods

Study population

The diagnosis and differential diagnosis of PHPT were made for all patients according to the current guidelines [1]. This retrospective study includes a total of 134 lesions (104 imaging-suspected parathyroid lesions and 30 concomitant thyroid nodules as a control group) of 101 patients with PHPT who underwent US-guided fine needle aspiration biopsy (FNAB) with PTH-WO performed according to the imaging findings between September 2020 and February 2023 in a tertiary referral center endocrinology, diabetes, and metabolic disease clinic. The medical history, demographic data, localization, tissue aspirate PTH assay results, laboratory results, type of surgery (if performed), pathological findings, and the postoperative course of the patients were recorded and analyzed.

Patients with positive, negative, or never scintigraphy were included in our study. All patients except one underwent PS, and all patients underwent detailed USG before the PTH-WO procedure, even if US had been performed previously. PS results were considered negative if there was no uptake of sestamibi on late images of the scintigraphy and suspicious if sestamibi uptake was confusing due to accompanying thyroid disease or if there was localization of uptake atypic for parathyroid lesion. According to the US findings, the patients were classified as negative (no lesion), suspicious (atypical US images, including shape, size, and echo), or positive (possible parathyroid lesion). A US-guided parathyroid FNA with PTH-WO was performed in each patient with positive, suspicious, or discrepant lesions revealed by the imaging results, either through sonography or scintigraphy. US-guided thyroid FNA with PTH-WO was performed in 30 simultaneous thyroid nodules, which were considered the negative control group. The accuracy of PTH-WO results of suspected parathyroid lesions was calculated according to the results of the operation. The definitive diagnosis of PA is confirmed postoperatively histopathologically.

Inclusion criteria

Patients aged 18 years and older diagnosed with PHPT were included in the study.

Exclusion criteria

Patients with tertiary hyperparathyroidism, familial hypocalciuric hypercalcemia, and PHPT due to familial multiple endocrine neoplasms were excluded.

Measurement of biochemical and hormonal parameters

The serum intact PTH levels were measured using an immunoassay method (Cobas c801, Roche Diagnostics, Indianapolis, IN) (normal PTH range: 15-65 ng/L). The serum 25-hydroxyvitamin D3 levels were measured using the high total electro-chemiluminescence method (Cobas c801, Roche Diagnostics). The serum total Ca, phosphorus (P), and albumin levels were measured using calorimetric methods (Cobas c702, Roche Diagnostics).

Technique for FNAB and PTH-WO

The skin was marked and cleaned after localizing the lesion. A 22-gauge needle was inserted into the suspicious neck lesion under US guidance for the procedure. After needle insertion, negative pressure was quickly applied to the suspicious lesion. After the sample was spread for cytological evaluation, the remaining portion was washed at least three times in a plastic tube containing 1 mL of normal saline, and the diluted aspirate was sent to the laboratory for PTH measurement [24]. No complications were recorded after the procedure. According to the ratio of PTH-WO levels to the patient’s serum PTH levels, patients were defined as PTH-WO positive (>1), according to the ratio of the PTH-WO levels to the upper limit of the laboratory PTH levels, patients were defined as PTH-WO negative (<1), and the values in between were proposed as gray zone.

Statistical analysis

IBM® Statistical Package for the Social Sciences (SPSS) version 20 (IBM® Corp., Armonk, NY) was used for data comparison. After the normal distribution was determined, an independent samples t-test was applied to normally distributed data, and a Mann-Whitney U test was conducted to compare non-normally distributed data. Pearson's chi-squared test was used to compare the ratios. The sensitivity was calculated as true positive/(true positive + false negative), specificity as true negative/(true negative + false positive), negative predictive value as true negative/(true negative + true positive), positive predictive value as true positive/(true positive + true negative) and accuracy as (true negative + true positive)/(true negative + true positive + false positive + false negative). A value of p < 0.05 was considered statistically significant.

## Results

In total, 101 patients (104 lesions) with PHPT underwent US-guided tissue FNA with PTH-WO. A total of 93 patients (93 lesions) had positive PTH-WO results. Eight patients (11 lesions) had negative PTH-WO results. In three of 101 patients, PTH-WO was performed from both suspicious lesions because PS and US pointed to two different sites. Eleven lesions with negative PTH-WO results but suspicious for PA on imaging findings were cytologically (three) and pathologically (eight) confirmed not to be PA, benign lymph nodes (n = 6), benign thyroid nodules (n = 4), and papillary thyroid cancer (n = 1).

In our study, five (4.95%) patients had unusual localization (three intra-thyroidal, one anterior mediastinal, and one jugular). Each lesion was diagnosed as PA using US-guided tissue FNA with PTH-WO.

The tables are lesion-based rather than patient-based. The mean age was 54.9 ± 12.0 years, and female predominance was observed (85 vs. 16). Table 1 shows the demographic data of the patients who underwent PTH-WO and a comparison between lesions with positive and negative tissue aspirate PTH tests is given in Table 2.

**Table 1:** Demographic, clinical, and laboratory parameters of the study group Ca: calcium; P: phosphorus. * Since it does not show normal distribution, it is given as a median and interquartile range of 25-75 percentiles (IQR).

Age (year)	54.9 + 12.0
Female/male (n)	77/16
Body mass index (kg/m^2^)	28.2 + 4.1
Ca (mg/dL)	11.84 + 1.41
P (mg/dL)	2.26 + 0.48
Parathyroid hormone (ng/L)	200, IQR (194.75)*
Urinary Ca (mg/day)	381.0 + 153.4
Creatinine (mg/dL)	0.78 + 0.26
Albumin (g/L)	43.6 + 3.4
T score	
Lumbar spine 1-4	-2.03 + 1.25
Femur total	-1.66 + 0.95

**Table 2:** Comparison between lesions with positive and negative tissue aspirate PTH tests PTH: parathyroid hormone; PTH-WO: PTH washout; Ca: calcium. * Since it does not show normal distribution, it is given as a median and interquartile range of 25-75 percentiles (IQR).

	PTH-WO (+) (n = 93)	PTH-WO (-) (n = 11)	p
Serum PTH (ng/L)	200.0, IQR (193.5)*	193.0, IQR (221.0)*	0.26
PTH-WO (ng/L)	5000.0, IQR (1600.0)*	17.0, IQR (13.1)*	<0.001
Ca(mg/dL)	11.8 + 1.5	11.8 + 1.0	0.96

In addition, PTH-WO was negative in all 30 thyroid lesions considered as negative controls and were comparable with the PTH-WO-negative group (p = 0.24). PTH-WO levels (median (IQR)) were significantly higher in the positive group (n = 93, 5000 (IQR: 1600) ng/L), compared to the negative group (n = 11, 17.0 (IQR: 13.1) ng/L) and concomitant thyroid nodule aspirate groups (n = 30, 14.0 (IQR: 4.3) ng/L) (p < 0.001). There was no correlation between PTH-WO concentrations and serum PTH and serum calcium levels.

Reliability of imaging methods and localization

**Figure 1:**
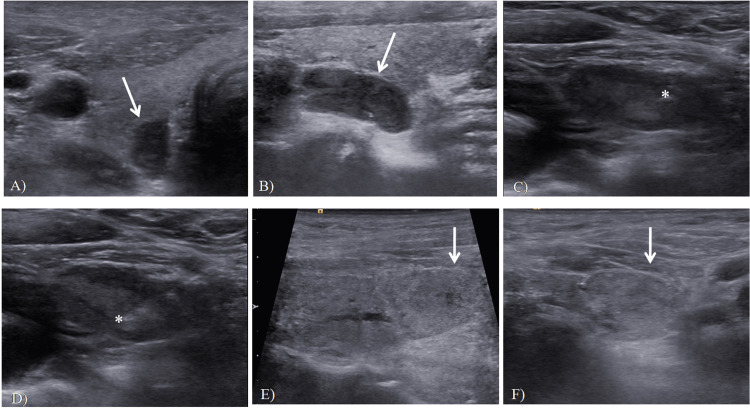
Hypoechoic lesion of 2 x 0.8 cm, which is suspected to be parathyroid adenoma, located in front of the trachea in the lower part of the isthmus of the right lobe of the thyroid (A, B). Transverse (A) and longitudinal (B) sections of the suspicious lesion. Hypoechoic lesion suspicious for a parathyroid adenoma, measuring 13 x 6 mm in the inferior part of the right lobe of the thyroid (C, D). Needle inserted into suspected lesion (C) and inside lesion (D). As shown in Figure E (longitudinal) and F (transverse), the lesions have the same echo as the thyroid gland and can be difficult to differentiate from thyroid nodules. Aspiration of the lesion was compatible with a parathyroid adenoma. * Needle tip.

Of the 100 patients who underwent PS, 49 (49%) had negative PS results. Of these 49 lesions that could not be detected on PS, 42 (85%) were detected by PTH-WO (Figure 1). Of the 13 lesions that could not be detected on initial US, 11 (84.6%) were detected by PTH-WO. Of the 104 lesions, 18 (6%) were triple positive, i.e., positive PS, positive US results, and positive tissue aspirate PTH-WO tests. Of the remaining 86 lesions that had suspicious or conflicting results between the PS and US, 75 were PTH-WO positive (72.1%) and 11 were PTH-WO negative (10.5%). In four of the 11 (36.3%) PTH-WO negative lesions, both PS and US mislocalized together.

One of the patients had no PS, but US-guided PTH-WO was positive; therefore, the data of the other 92 positive patients (103 lesions) are shown in Table 3. Comparisons between the results of PTH-WO and localization studies are shown in Table 3.

**Table 3:** PTH-WO distributions of parathyroid scintigraphy, ultrasonography 1, and ultrasonography 2 PTH: parathyroid hormone; PTH-WO: PTH washout.

		Parathyroid scintigraphy					
		Positive (n = 34)		Suspicious (n = 20)		Negative (n = 49)	
		PTH-WO (+) (n = 30)	PTH-WO (-) (n = 4)	PTH-WO (+) (n = 20)	PTH-WO (-) (n = 0)	PTH-WO (+) (n = 42)	PTH-WO (-) (n = 7)
Ultrasonography 1	Positive (n = 66)	18	3	10	0	31	4
	Suspicious (n = 24)	8	1	6	0	8	1
	Negative (n = 13)	4	0	4	0	3	2
Ultrasonography 2	Positive (n = 94)	18	2	10	0	31	0
	Suspicious (n = 9)	12	2	10	0	11	7

The sensitivity, specificity, negative predictive value, and positive predictive value of PS, US, and US-guided PTH-WO methods as confirmatory diagnostic methods for PA are presented in Table 4.

**Table 4:** Predictive values of PS, US1, and US2 + PTH-WO PS: parathyroid scintigraphy; US: ultrasonography; PTH: parathyroid hormone; PTH-WO: PTH washout; NPV: negative predictive value; PPV: positive predictive value.

	Sensitivity	Specificity	NPV	PPV	Accuracy
PS	0.42	0.64	0.19	0.81	0.45
US1	0.64	0.36	0.06	0.94	0.98
US2 + PTH-WO	1.00	0.82	0.09	0.91	0.82

Surgical intervention

A total of 95 patients (90 FP and five BNE) were operated. Of the 93 PTH-WO positive patients, 91 (90 FP and one BNE) were successfully operated, and pathological evaluation of the surgical specimen confirmed that 91 patients (100%) were PA. Two PTH-WO-positive patients with cystic parathyroid lesions were not operated, as they went into remission after aspiration of the cyst with PTH-WO. One patient with a PTH-WO-positive lesion underwent BNE because the other accompanying suspicious lesion was PTH-WO negative (PS finding and US localization did not match) and four patients with PTH-WO-negative lesions underwent BNE. All PTH-WO-positive lesions were pathologically proven to be parathyroid tissue (except for two cystic lesions) and eight PTH-WO-negative lesions were pathologically proven not to be parathyroid tissue. One patient with a negative PTH-WO result underwent unsuccessful surgery despite BNE with intraoperative PTH measurement at another health institution. Surgery was not performed in three patients due to controllable PTH levels and patient preferences. In surgically treated lesions, the positive predictive value of tissue aspirate PTH tests is 100%.

## Discussion

Our study reveals that US-guided tissue PTH-WO is a more reliable method for the localization of PA than US and PS alone. The US-guided PTH-WO results significantly reduced the number of patients requiring BNE.

Accurate localization in patients with PHPT decreases unnecessary BNE [5,26,27]. FP has many advantages over BNE [5,28,29]. Since the cause of PHPT is mostly (90%) solitary PA, FP is sufficient for most patients [5,30,31]. Our results showed that a strong preoperative localization improves FP. At least 11 of 49 PS-negative patients and seven of 13 first US-negative patients would have needed BNE if PTH-WO had not been performed. PTH-WO helped identify the potential PAs. Thus, our study findings reveal that PTH-WO improves FP, which aligns with previous studies' findings [23-25,32]. From another perspective, combining localization studies with PTH-WO measurement decreases the risk of surgical failure by avoiding false-negative and false-positive results. In the present study, the PS findings were false-positive in four patients, and the initial US findings were false-positive in seven patients, which were detected by PTH-WO measurements during the preoperative period. Conversely, although IO-PTH tests have become available since 1996 if there is no preoperative prediction, the patient will most likely go for BNE [33,34]. In many centers, like our center, it is not possible to perform IO-PTH analysis. Thus, preoperative PA localization can be confirmed using the PTH-WO method, but certain aspects still need improvement.

Although tissue FNA with PTH-WO is a clinically useful and straightforward method, there is no established standard for the PTH level as positive or negative evidence of whether the aspirated tissue is parathyroid. As no other tissue contains PTH, the PTH level in non-parathyroid tissues should be undetectable, and the PTH assay should be regarded as positive even if modest levels of the hormone are found, according to Sacks et al. [35]. However, low PTH levels were found in most instances, likely due to blood contamination [35]. The present study detected PTH levels in all non-parathyroid lesions (benign-malignant thyroid nodules and lymph nodes), as proven by cytological or histopathological examination. In non-parathyroid lesions, regardless of the patient's serum PTH levels, PTH-WO levels were low, with the majority even being close to the lower limit of the normal laboratory value, according to our findings. However, a few were measured within the normal laboratory range. For the negative tissue aspirate, we advise keeping the rate of the PTH-WO levels to the upper limit of the laboratory PTH levels under 1. Accepting a lower value as a threshold for considering the lesion as negative would raise the rate of false positivity when we take such instances into account using the sampling technique. The levels lower than the patient's serum PTH level and higher than the upper limit of laboratory PTH level can be considered a gray zone, and PTH-WO should be repeated. The most important gap here seems to be determining values that would be considered negative. Further studies on this subject are warranted.

Naturally, each patient has a different serum PTH level in PHPT; sometimes, only a level slightly above the upper limit of the laboratory or very high levels may represent PHPT. Therefore, giving a positive cut-off value for PTH-WO based on the serum PTH level is not reasonable. Among the studies presenting a fixed cut-off value, Canpolat et al. [36] reported a level greater than 436.5 ng/L for FNAB PTH-WO with a sensitivity and specificity of 90.3% and 88.9%, respectively. Frasoldati et al. [37] washed tissue aspirates with 1 mL saline and reported a sensitivity and specificity of 100%, with a cut-off level of 101 ng/L. In the present study, tissue aspirates were washed with 1 mL saline; in most cases, the PTH-WO levels were >5000 ng/L in the first measurement and exceeded hundreds of thousands upon dilution. Therefore, we limited the upper limit to 5000 ng/L in our study, as it would not contribute further. The lowest PTH-WO level was more than two times higher than the patient's serum PTH level. If we had adapted the present study to the study of Canpolat et al. [36], which used a fixed cut-off value of 436 ng/L, false negative results would have increased in patients with low serum PTH levels and we would have missed true positive patients. On the other hand, if we lower the PTH-WO threshold, which will be accepted as a positive test result, to fixed levels below the serum PTH levels of the patients, as in Frasoldati et al. [37], the number of unsuccessful surgeries may increase. This problem needs to be supported by additional studies.

Maser et al. [38] also washed the tissue aspirated with 1 mL saline and reported that a value above the normal range (6-40 pg/mL) indicated parathyroid tissue and a 49-65 pg/mL value was considered suspicious. From another viewpoint, the first sentence indicates the cut-off value that we consider negative. In light of our data, we think that a negative PTH-WO level will not exceed the laboratory PTH limits.

Abdelghani et al. [32] washed the aspirate in 2 mL saline and reported a sensitivity of 91.6% and a positive predictive value of 100% when the tissue aspirate PTH level was higher than the serum PTH level at aspiration. Hung et al. [25] washed the tissue aspirate in 6 mL saline and estimated the aspirate volume to be no more than 0.2 mL. Therefore, a positive result was considered when the tissue aspirated PTH level was more than one-thirtieth of the serum PTH level (0.2/6 mL). Obołończyk et al. [39] recommend that a PTH-WO equal to or greater than the serum PTH level is a reasonable cut-off value and presented the potential interpretation of a lower PTH-WO PTH concentration/serum PTH concentration ratio (0.5-1). Özderya et al. [24] defined PTH levels higher than serum PTH levels PTH-WO as positive. Sacks et al. [35] washed the tissue aspirate in 0.25 mL saline and most positive values were unequivocal, often hundreds to thousands of picograms per milliliter. If we adapt the current study to the study of Hung et al. [25], in our study, tissue aspirate was washed in 1 mL saline. Assuming that the maximum aspirate volume was 0.2 mL, if we had accepted a tissue aspirate PTH level higher than one-fifth of the serum PTH level (0.2 mL/1 mL) as a positive result, we could have interpreted the true negative results of our 11 patients with serum PTH levels <105 ng/L as false positives in accordance with the study of Hung et al. [25]. Our study included only PHPT cases, whereas the study of Hung et al. [25] included patients with tertiary hyperparathyroidism. The higher serum PTH levels of patients with tertiary hyperparathyroidism may have prevented false positives in their study.

In the present study, we found that PTH levels in PAs were highly concentrated and only a certain amount was released into the circulation, independent of the serum Ca and PTH levels of the patients. Unfortunately, we do not know what determines this concentration and why it is concentrated two-fold in some and 1000-fold in others. Since we cannot predict the PTH level in the adenoma, a level based on a positive cut-off value or based on dilution would not be appropriate in most cases of PHPT. Our recommendation is to consider positive a PTH-WO level > patient’s serum PTH level in aspirate washed with 1 ml saline. This positive assay definition sufficiently confirms the diagnosis and does not allow negative lesions to be missed. Our results strongly support previous studies demonstrating the value of this technique. The strengths of our study are the high number of surgically confirmed positive cases compared to the general literature.

Clinically, it is crucial to refrain from needless therapies for possibly true negative lesions when managing PHPT. Although no lesions entering the gray area were found in our study, it is still critical to re-examine problematic cases entering a possible gray area. The inclusion of 30 simultaneous thyroid aspirates as a control group and the number of patients who could be considered definitively negative is one of the strengths of the present study. We would like to emphasize that we did not detect false positives in any of these 30 control groups. In this respect, this study may contribute to filling the gaps in the literature.

Furthermore, this study is one of the rare ones demonstrating the complexity and contradictions of imaging techniques in PHPT. Our study demonstrated that even in difficult cases with negative or discordant localization results, PTH-WO, which can be performed in most local centers, can be used as a bridge. The use of 4D-CT in PA localization, which has been represented as allowing minimally invasive treatments, does not always produce reliable results, and false-positive localization is frequent [40].

Since the diagnosis of parathyroid lesions is often impossible, with FNAB alone, the positive PTH-WO results of a suspected parathyroid lesion can be used as solid evidence to allow non-surgical treatments, such as RFA [6,7]. Thus, with this quick and reliable method of localizing PAs and confirming localization, both diagnosis and treatment can be performed in the same session. In patients who are poor candidates for surgery, in high-risk groups, or in the geriatric population, minimally invasive treatments can easily be used with this highly reliable method. Our study's primary limitation is that it is retrospective in nature. We must first define a potential PA and show accessible sampling locations, which is a key drawback of PTH-WO with FNAB.

## Conclusions

For separating parathyroid lesions from other cervical lesions, PTH-WO combined with US is more accurate than traditional imaging methods. Our results suggest that PTH-WO can be used as an easy-to-implement and reliable guide to increasing FPs, especially when imaging modalities are inadequate and in centers where 4D-CT and intra-operative PTH are not available. This technique, which satisfactorily verifies the diagnosis, will likely be employed very soon to enable the non-surgical treatment of PAs.
